# Neurofilament Light Chains in Serum Predict Post—Transjugular Intrahepatic Portosystemic Shunt Hepatic Encephalopathy

**DOI:** 10.1002/mco2.70475

**Published:** 2025-11-05

**Authors:** Christian Labenz, Eva Maria Schleicher, Myriam Meineck, Martin A. Kabelitz, Alena F. Ehrenbauer, Anja Tiede, Jim B. Mauz, Sven Danneberg, Michael Bernhard Pitton, Falk Steffen, Julia Weinmann‐Menke, Peter Robert Galle, Stefan Bittner, Felix Lüssi, Jens Uwe Marquardt, Benjamin Maasoumy, Simon Johannes Gairing

**Affiliations:** ^1^ Department of Internal Medicine I University Medical Center of the Johannes Gutenberg‐University Mainz Germany; ^2^ Cirrhosis Center Mainz (CCM) University Medical Center of the Johannes Gutenberg‐University Mainz Germany; ^3^ Research Center for Immunotherapy (FZI) University Medical Center of the Johannes Gutenberg‐University Mainz Germany; ^4^ Department of Gastroenterology Hepatology, Infectious Diseases and Endocrinology, Hannover Medical School Hannover Germany; ^5^ Department of Medicine I University Hospital Schleswig‐Holstein Lübeck Germany; ^6^ Department of Radiology University Medical Center of the Johannes Gutenberg‐University Mainz Germany; ^7^ Department of Neurology University Medical Center of the Johannes Gutenberg‐University Mainz Germany

**Keywords:** axonal damage, biomarkers, cognitive dysfunction, hepatic encephalopathy, post‐TIPS HE, transjugular intrahepatic portosystemic shunt

## Abstract

Hepatic encephalopathy (HE) after transjugular intrahepatic portosystemic shunt (TIPS) insertion constitutes a frequent and severe complication. However, there is a lack of robust predictive biomarkers for post‐TIPS HE, so far. This study evaluated the usefulness of neurofilament light chains (NfL) and glial fibrillary acidic protein (GFAP) in serum for predicting post‐TIPS HE. Around 144 patients with cirrhosis from three centers were prospectively included and monitored for the occurrence of post‐TIPS overt HE, liver transplantation, and death. Serum NfL and GFAP were evaluated before TIPS insertion using the single molecule array technology. In a subgroup of patients sequential NfL and GFAP levels were assessed at 30‐ and 180‐days post‐TIPS. While higher NfL levels (sHR 1.01, *p* = 0.036) were independently associated with post‐TIPS OHE after adjusting for other risk factors, GFAP levels had no predictive ability. Consistently, only elevated NfL levels were associated with a higher risk of death/liver transplantation in multivariable analyses. Sequential measurements of NfL at 30 and 180 days after TIPS revealed that NfL levels remain constant until Day 30 followed by a decrease at day 180. Notably, GFAP levels did not change over time. Thus, NfL could be a valuable biomarker for identifying high‐risk patients for post‐TIPS HE.

## Graphical abstract

Hepatic encephalopathy (HE) after transjugular intrahepatic portosystemic shunt (TIPS) insertion constitutes a frequent and severe complication, however, biomarkers are currently scarce. The results of this study indicate that neurofilament light chains (NfL) in serum, but not glial fibrillary acidic protein (GFAP), is a useful biomarker identifying patients at a higher risk for post‐TIPS HE. In the future, NfL might serve as a promising biomarker to improve personalized management of patients receiving a TIPS.

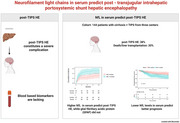

## 1 Introduction

Portal hypertension is one of the main contributors to the development of complications in liver cirrhosis [1]. The insertion of a transjugular intrahepatic portosystemic shunt (TIPS) is an effective treatment for most portal hypertension‐related complications, such as ascites or variceal hemorrhage [2]. In addition, TIPS has also been linked to an improved prognosis [3].

Although interdisciplinary pre‐interventional work‐up as well as placement techniques for TIPS implantation improved over the last few decades, hepatic encephalopathy (HE) continues to be one of the most common and significant complications post‐TIPS [4]. In general, the risk of developing overt post‐TIPS HE is approximately 30%–50% and predominantly observed within the first few months after implantation [4]. Despite the considerably high risk of post‐TIPS HE, current guidelines do not recommend prophylactic treatment, such as lactulose or rifaximin, for all patients after TIPS insertion [5] and Rifaximin may only be considered in patients with a history of OHE [5, 6]. Therefore, predicting post‐TIPS HE, especially in patients without an OHE history, remains challenging.

Identifying high‐risk groups for post‐TIPS HE would be desirable to enable the implementation of personalized preventive strategies and also in order to identify patients that might not be suitable for TIPS treatment. Several lines of evidence demonstrate that clinical parameters, such as older age, a lower portosystemic gradient after TIPS insertion, a higher MELD score, or sarcopenia, are associated with a higher risk of HE [4, 7].

So far, blood‐based biomarkers for predicting post‐TIPS HE are scarce [8]. Recently, glial fibrillary acidic protein (GFAP), an intermediate filament found in astrocytes, and especially neurofilament light chains (NfL), an important structural protein found in neurons, have emerged as promising biomarkers for HE in patients with cirrhosis [9, 10]. However, while both molecules have mostly been assessed in cross‐sectional studies that differentiate patients with various degrees of HE or without HE [9, 10, 11, 12, 13], their relevance as biomarkers for post‐TIPS HE has not been evaluated, yet.

Therefore, the aim of this study was (i) to assess whether NfL and GFAP serum levels are associated with the development of post‐TIPS HE and, (ii) to investigate the course of NfL and GFAP levels in patients with cirrhosis after TIPS.

## 2 Results

This study included 144 patients with a TIPS insertion for refractory ascites or secondary prophylaxis after variceal bleeding (Mainz: *n* = 57, Hannover: *n* = 71, Lübeck: *n* = 16) (Figure S1). Baseline characteristics are displayed in Table 1. The cohort was predominantly male with a median age of 61 years (IQR 53, 67) and a median MELD of 12 (IQR 9, 14). Seven patients (5%) were in the FIPS high‐risk group (≥0.92). The leading etiology of cirrhosis was alcoholic liver disease (ALD). Of note, no patients with ongoing alcohol consumption received a TIPS in this cohort. At study inclusion prior to TIPS insertion, 72 patients (58%) had pathological results in PHES (CHE+). A total of 40 patients (28%) had a history of OHE. Of note, all of these events had a trigger that could be eliminated. Median NfL and GFAP serum levels in the total cohort were 39 pg/mL (IQR 23, 65) and 187 pg/mL (IQR 117, 298), respectively. Comparisons between patients with NfL or GFAP levels above and below the respective medians are presented in Tables S1 and S2.

**TABLE 1:** Demographics and baseline characteristics of the study cohort.

Variable	*N*	*N* = 144
Age (years)	144	61 (53, 67)
Sex	144	
male		99 (69%)
female		45 (31%)
Indication for TIPS	144	
Refractory ascites		103 (72%)
Sec. prophylaxis after variceal bleeding		34 (24%)
Other		7 (4.9%)
Etiology of cirrhosis	144	
ALD		70 (49%)
MetALD		8 (6%)
MASLD		20 (14%)
Viral		8 (6%)
Other		30 (21%)
ALD + viral		8 (6%)
PSG delta (%)	144	59 (49, 69)
TIPS diameter (mm)	144	
6		33 (23%)
7		3 (2.1%)
8		74 (51%)
10		34 (24%)
CHE (PHES)	124	
CHE−		52 (42%)
CHE+		72 (58%)
PHES	123	−5.0 (−8.0, −2.0)
Child Pugh	144	
A		21 (15%)
B		108 (75%)
C		15 (10%)
FIPS score	144	−0.18 (−0.64, 0.36)
MELD score	144	11.8 (9.1, 14.4)
History of OHE	144	40 (28%)
Sodium (mmol/L)	142	136.0 (133.0, 138.0)
Creatinine (mg/dL)	144	1.09 (0.86, 1.52)
Bilirubin (mg/dL)	144	1.08 (0.73, 1.59)
Albumin (g/L)	144	31.0 (27.5, 36.5)
CRP (mg/L)	142	9 (4, 16)
WBC (per nL)	143	5.10 (3.70, 7.50)
Hemoglobin (g/dL)	143	10.10 (8.80, 11.70)
Platelets (per nL)	142	111 (70, 173)
Ammonia pre‐TIPS (ULN)	126	0.72 (0.57, 0.92)
NfL (pg/mL)	144	39 (23, 65)
GFAP (pg/mL)	144	187 (117, 298)
Lactulose use	144	82 (57%)
Rifaximin use	144	69 (48%)

*Note*: Data are expressed as median with interquartile range or as number with percentages.

Abbreviations: ALD, alcoholic liver disease; CHE, covert hepatic encephalopathy; GFAP, glial fibrillary acidic protein; MASLD, metabolic dysfunction‐associated steatotic liver disease; MELD, model for end‐stage liver disease; MetALD, metabolic and alcoholic liver disease; NfL, neurofilament light chains; OHE, overt hepatic encephalopathy; PHES, psychometric hepatic encephalopathy score; PSG, portosystemic gradient.

### 2.1 NfL/GFAP Levels and Development of Post‐TIPS OHE

All 144 patients were followed for the development of OHE, liver transplantation and/or death after TIPS insertion. The median follow‐up time until censoring, death or liver transplantation was 289 days (IQR 108, 620). During the observational period, 55 (38%) patients developed at least one OHE episode. In addition, 43 (30%) patients died or received a liver transplantation. Of these, 20 (14%) died or received a liver transplantation without developing OHE prior to these events. Of the patients with an OHE episode, 39 (71%) had HE grade II, 15 (27%) HE Grade III and 1 (2%) HE Grade IV. Most OHE events occurred during the first 3 months after TIPS insertion. The density plot describing the time points of the occurrence of the OHE episodes is displayed in Figure S2.

In univariable Fine and Gray analyses, higher NfL serum levels were associated with the development of a post‐TIPS OHE episode (sHR 1.01, 95% CI 1.00–1.01, *p* < 0.001), while GFAP serum levels were not. Other variables significantly associated with post‐TIPS OHE in univariable analyses were a higher MELD score, a higher FIPS score, higher creatinine levels and lower platelets (Table 2).

**TABLE 2:** Univariable fine and gray regression analysis for OHE development.

Characteristic	N	sHR	95% CI	*p*‐value
Age (years)	144	1.02	1.00, 1.04	0.11
Female sex	144	0.90	0.49, 1.67	0.8
PSG delta (%)	144	1.00	0.99, 1.02	0.6
TIPS diameter (mm)	144	1.18	0.98, 1.42	0.078
MELD	144	1.07	1.00, 1.13	0.035
FIPS	144	1.42	1.01, 1.99	0.045
History of OHE	144	1.34	0.74, 2.40	0.3
CHE	124	1.85	0.99, 3.45	0.053
PHES	124	0.96	0.90, 1.03	0.3
Sodium (mmol/L)	142	1.00	0.94, 1.07	0.9
Creatinine (mg/dL)	144	1.46	1.22, 1.74	<0.001
Bilirubin (mg/dL)	144	0.97	0.72, 1.31	0.9
INR	144	1.62	0.52, 5.09	0.4
Albumin (g/L)	144	0.98	0.94, 1.01	0.2
CRP (mg/L)	142	1.01	1.00, 1.02	0.4
WBC (per nL)	143	0.98	0.88, 1.08	0.7
Hemoglobin (g/dL)	143	0.87	0.74, 1.01	0.069
Platelets (per nL)	142	1.00	0.99, 1.00	0.025
Ammonia (/ULN)	126	1.64	0.89, 3.03	0.11
NfL (pg/ml)	144	1.01	1.00, 1.01	0.031
NfL above the median	144	1.92	1.12, 3.29	0.018
GFAP (pg/ml)	144	1.00	1.00, 1.00	0.3
GFAP above the median	144	1.10	0.65, 1.85	0.7
Lactulose	144	0.93	0.55, 1.57	0.8
Rifaximin	144	0.91	0.54, 1.54	0.7

In univariable Fine and Gray regression for OHE development, liver transplantation and death were treated as competing events.

Abbreviations: CHE, covert hepatic encephalopathy; CI, confidence interval; CRP, c‐reactive protein; FIPS, Freiburg index for post‐TIPS survival; OHE, overt hepatic encephalopathy; GFAP, glial fibrillary acidic protein; MELD, model for end‐stage liver disease; NfL, neurofilament light chains; PHES, psychometric hepatic encephalopathy score; PSG, portosystemic gradient; sHR, subdistribution hazard ratio; WBC, white blood cell count.

**FIGURE 1:**
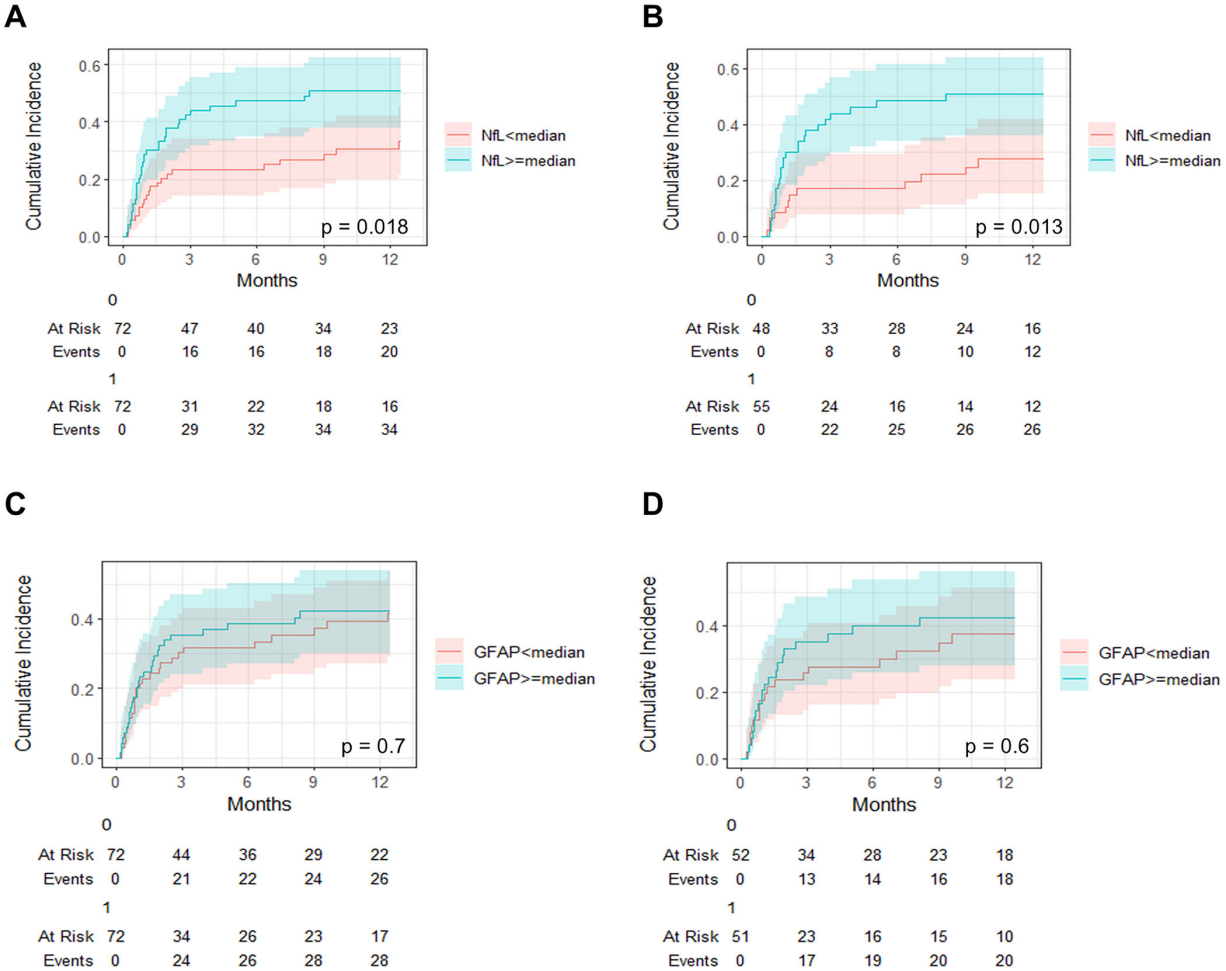
Cumulative overt HE (OHE) incidence stratified by NfL or GFAP medians. Cumulative OHE incidence in (A) patients stratified by the NfL median or (C) GFAP median in the total cohort. Cumulative OHE incidence in (B) patients stratified by the NfL median or (D) GFAP median in the subcohort of patients with ascites as indication for TIPS insertion. 0: < median; 1: ≥ median.

When stratifying the cohort according to the NfL median, patients with NfL levels above the median had a significantly higher cumulative OHE‐incidence than patients with NfL levels below the median (*p* = 0.018, Figure 1A). In contrast, patients with GFAP levels above the median had a comparable OHE‐incidence as patients with GFAP levels below the median (*p* = 0.7, Figure 1C). To identify variables independently associated with the development of OHE and to further analyze NfL and GFAP serum levels in this context, we fitted various multivariable regression models using the method of Fine and Gray. For this purpose, we fitted regression models including the univariable significant variables (MELD and platelets), established risk factors (history of OHE, PSG delta in % and age) as well as NfL or GFAP levels. Of note, creatinine, and FIPS score were not included in this analysis due to collinearity with MELD. NfL levels as a metric variable were independently associated with OHE development, while GFAP was not (Table 3).

**TABLE 3:** Multivariable Fine and Gray regression analysis for OHE development.

Characteristic	N	sHR	95% CI	*p*‐value
**Model 1**				
NfL (pg/mL)	142	1.01	1.00, 1.01	0.028
PSG delta (%)	142	1.00	0.98, 1.02	0.8
MELD	142	1.05	0.99, 1.12	0.11
History of OHE				
0	102	—	—	
1	40	1.14	0.62, 2.11	0.7
Platelets	142	1.00	0.99, 1.00	0.028
Age	142	1.02	0.99, 1.04	0.2
				
**Model 2** ^a^				
NfL above the median	142	1.74	0.93, 3.26	0.083
				
**Model 3** ^a^				
GFAP (pg/mL)	142	1.00	1.00, 1.00	0.2
				
**Model 4** ^a^				
GFAP above the median	142	1.01	0.57, 1.81	0.9

In multivariable Fine and Gray regression analysis for OHE development, liver transplantation and death were treated as competing events.

Abbreviations: CI, confidence interval; GFAP, glial fibrillary acidic protein; MELD, model for end‐stage liver disease; NfL, neurofilament light chains; OHE, overt hepatic encephalopathy; PSG, portosystemic gradient; sHR, subdistribution hazard ratio.

^a^ Models were adjusted for PSG delta (%), MELD, history of OHE, platelets, and age.

In addition, we repeated the analyses including lactulose/rifaximin use and also conducted separate analyses including FIPS instead of MELD and age (Tables S3, S4, and S5). Moreover, it is well‐known that NfL levels can be influenced by renal function. Therefore, we also repeated the multivariable analyses including creatinine and bilirubin instead of MELD (Table S6). In all these aforementioned analyses, NfL remained independently associated with OHE development.

Given that all patients with a history of OHE were treated with a secondary prophylaxis and might differ in terms of prognosis compared to patients without a history of OHE, we also conducted a subgroup analysis in the patients without a history of OHE prior to TIPS (*n* = 102 with all variables available). Again, NfL remained independently associated with a higher risk of post‐TIPS OHE (Table S7).

Next, we analyzed non‐linear effects of NfL on the risk of post‐TIPS HE. Here, NfL levels showed a lower relative hazard with a linear relation until 60 pg/ml with a subsequent flattening of the curve (Figure S3A). Non‐linear effects of GFAP on the risk of post‐TIPS HE are displayed in Figure S3B. Of note, NfL serum levels modeled as a restricted cubic spline with four knots (HR 2.12, 95 % CI 1.14, 3.95) remained independently associated with a higher risk of post‐TIPS HE in a multivariable model after adjusting for MELD and age. We did not include other variables into the model to avoid overfitting due to the limited number of outcome events.

The predominant subgroup of our cohort had refractory ascites as indication for TIPS insertion (*n* = 103, 72%). Given that these patients might differ from patients with TIPS for secondary prophylaxis after variceal bleeding, we repeated the analyses in this subgroup. Here, cumulative OHE incidence remained higher in patients with NfL serum levels above the median compared to below the median (Figure 1B). Again, higher GFAP levels did not reach significance (Figure 1D). In multivariable Fine and Gray regression analyses, NfL serum levels (sHR 1.01, 95% CI 1.00–1.01, *p* < 0.001) remained independently associated with the development of post‐TIPS HE, while GFAP was not (*p* = 0.3) (Table S8 and S9).

### 2.2 NfL/GFAP Levels and Death/Liver Transplantation After TIPS Insertion

In the total cohort, 31 (22%) patients died and 12 (8%) underwent liver transplantation. For the subsequent analyses, we investigated the potential association between NfL and GFAP serum levels and transplantation‐free survival.

**FIGURE 2:**
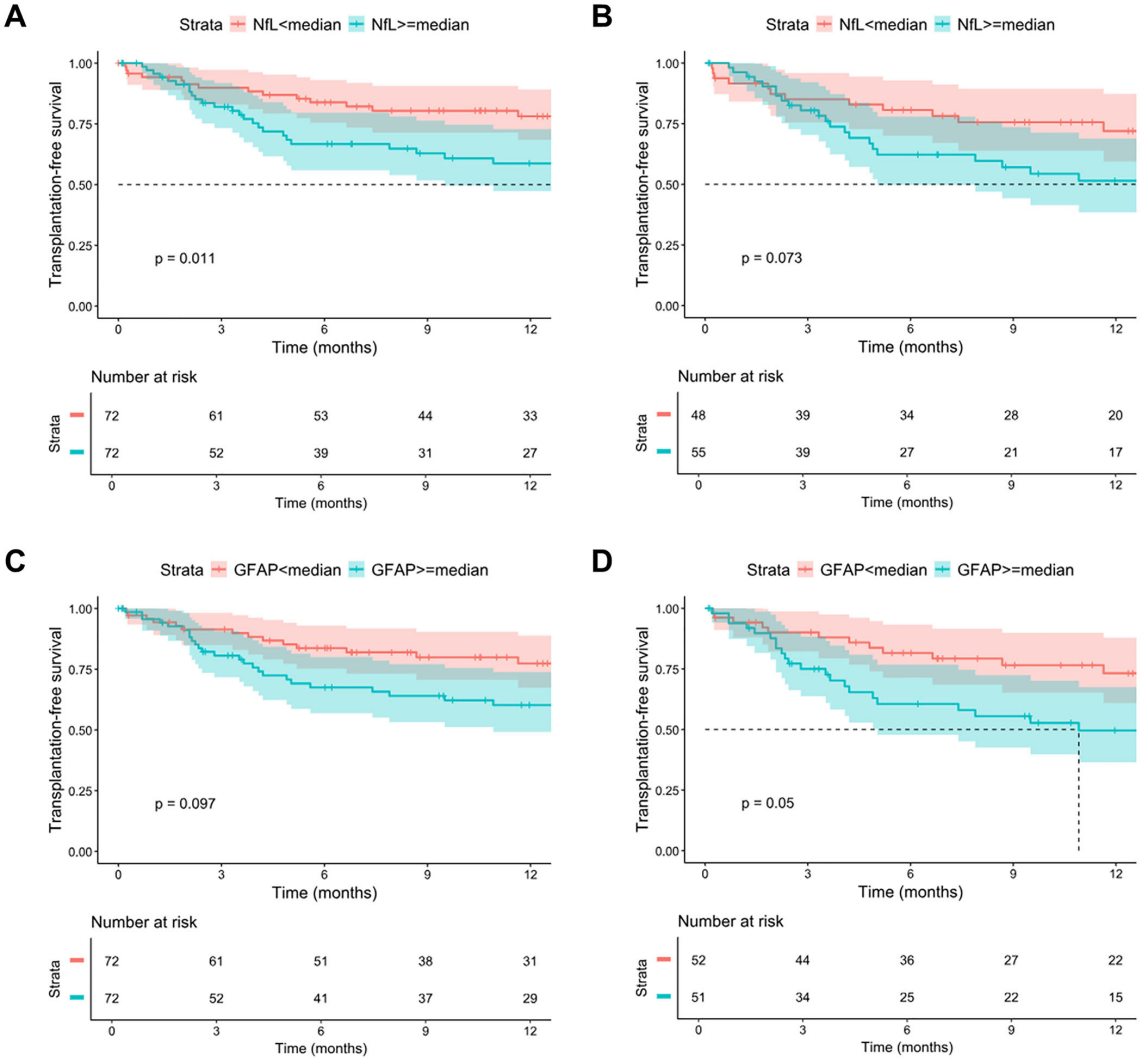
Liver transplantation‐free survival stratified by NfL or GFAP medians. Liver transplantation‐free survival in (A) patients stratified by the NfL median or (C) GFAP median in the total cohort. Liver transplantation‐free survival in (B) patients stratified by the NfL median or (D) GFAP median in the subcohort of patients with ascites as indication for TIPS insertion. 0: < median; 1: ≥ median.

In univariable Kaplan–Meier plots, transplantation‐free survival was significantly better in patients with NfL below the respective median in the total cohort (log‐rank *p* = 0.011), while prognosis did not differ between patients stratified according to GFAP median (log‐rank *p* = 0.097; Figure 2A,C). Univariable analyses in the subgroup of patients with ascites as indications for TIPS are displayed in Figure 2B,D. In univariable Cox regression analyses, higher age, a higher MELD score, a higher FIPS score, a history of OHE, lower sodium, higher creatinine, lower albumin, lower hemoglobin, and higher NfL serum levels were associated with a higher risk of death or liver transplantation (Table S10). Due to the limited number of outcome events, we did not include all univariable significant variables in one multivariable Cox regression model to avoid overfitting. Therefore, we decided to build models including age, MELD as well as NfL or GFAP levels. Here, NfL levels were independently associated with a higher risk of reaching the composite endpoint of death/liver transplantation, either as a metric variable (HR 1.01, 95% CI 1.00–1.01, *p* = 0.027) or when dichotomized according to the median (HR 1.95, 95% CI 1.03–3.67, *p* = 0.039) (Table 4). In contrast, GFAP was not associated with death/liver transplantation during follow‐up (Table 4).

**TABLE 4:** Multivariable Cox regression analysis for death/liver transplantation.

Characteristic	N	HR	95% CI	*p*‐value
**Model 1**				
NfL (pg/mL)	144	1.01	1.00, 1.01	0.027
MELD	144	1.17	1.09, 1.25	<0.001
Age (years)	144	1.04	1.01, 1.07	0.022
				
**Model 2** ^a^				
NfL above the median	144	1.95	1.03, 3.67	0.039
				
**Model 3** ^a^				
GFAP (pg/mL)	144	1.00	1.00, 1.00	0.4
				
**Model 4** ^a^				
GFAP above the median	144	1.32	0.70, 2.50	0.4

Abbreviations: CI, confidence interval; GFAP, glial fibrillary acidic protein; HR, hazard ratio; MELD, model for end‐stage liver disease; NfL, neurofilament light chains.

^a^ Models were adjusted for MELD and age.

### 2.3 Trajectory of NfL and GFAP Levels After TIPS Insertion

A strong correlation between NfL and GFAP (rho = 0.56) was observed at baseline. Additionally, levels of both NfL (rho = ‐0.39) as well as GFAP (rho = ‐0.30) inversely correlated with PHES.

In a subgroup of the patients from Mainz, follow‐up measurements of NfL and GFAP serum levels were available 30 days (n = 35), and 180 days (n = 21; 6 with OHE during the complete follow‐up) after TIPS insertion. Of these, 16 patients had available measurements at all three time points (prior to TIPS, after 30 days and after six months).

The trajectory of NfL and GFAP levels within 30 days as well as 180 days after TIPS insertion is displayed in Figure S4. In brief, NfL levels significantly decreased after six months when compared to baseline (‐10.9 pg/ml, 95% CI ‐20.7 ‐ ‐1.1, p = 0.001), while there was no significant change in NfL levels between baseline and day 30 after TIPS (p = 0.7). GFAP levels did not change significantly during follow‐up (Figure S4). There was no significant difference in the delta of NfL as well as GFAP serum levels from baseline to six months post TIPS when comparing patients with or without an OHE episode during follow‐up (Figure S5). There was a strong correlation between changes (delta of level at 180 days minus level pre‐TIPS) of NfL and GFAP within 180 days post‐TIPS (Figure S6). Additionally, there was a relevant positive correlation between delta NfL as well as delta GFAP and delta IL‐6 as well as delta CRP. In contrast, there was an inverse correlation between delta NfL as well as delta GFAP and delta ammonia. Of note, only delta GFAP had a relevant inverse correlation with delta PHES, while delta NfL did not (Figure S6).

## 3 Discussion

Blood‐based biomarkers for predicting post‐TIPS HE are needed to pave the way for personalized preventive strategies. In this multicenter study, we found that higher serum NfL levels prior to TIPS insertion are associated with both post‐TIPS OHE and transplantation‐free survival in patients with cirrhosis. This finding was confirmed in a subgroup analysis only including patients with ascites as indication for TIPS insertion. In contrast, GFAP serum levels were not independently associated with both outcome events. In addition, we provide insight into the longitudinal trajectory of NfL and GFAP serum levels after TIPS insertion in a sub‐cohort. Here, NfL levels decrease from baseline to six months of follow‐up, while GFAP levels remain unchanged.

At baseline prior to TIPS insertion, we found a moderate, but significant negative correlation of NfL and GFAP levels with PHES. In line, the prevalence of CHE was higher in patients with NfL or GFAP levels above the median of the cohort. These findings confirm the previously published association between higher NfL/GFAP levels and CHE and also the robustness and validity of our data [9, 10, 13].

The main finding of this study was the strong association between higher NfL serum levels and the development of post‐TIPS OHE as well as poorer transplant‐free survival, while GFAP was not predictive. This is the first study investigating NfL and GFAP for predicting OHE episodes after TIPS and, therefore, no direct comparison is available. However, there are studies available demonstrating the usefulness of NfL serum levels for detecting patients with and without OHE in a cross‐sectional setting [12, 13]. All in all, our findings may be of importance for future personalized preventive treatment strategies for patients receiving a TIPS by adding a new blood‐based biomarker in NfL to other established risk factors, such as poorer liver function or sarcopenia [4].

Due to the design of our study, we are unable to provide mechanistic insights into the question why NfL levels are associated with a higher risk of post‐TIPS HE. However, we hypothesize that higher NfL serum levels might be an indicator for a lower cognitive resilience, most likely due to prior (subclinical) damage, resulting in a greater susceptibility to metabolites and stressors causing HE after TIPS.

In the subgroup of patients with NfL and GFAP levels available at one and six months after TIPS, we found a relevant decrease in NfL levels after six months, while GFAP levels did not change. When interpreting these findings, one has to keep in mind that selection bias might be present, because patients had to be alive without liver transplantation to attend the follow‐up after six months. Therefore, these findings only apply to patients with a good prognosis after TIPS. Nevertheless, we speculate that the decrease of NfL levels in these patients might reflect that axonal damage decreases during the follow‐up period. Of note, it is an interesting finding that both NfL and GFAP changes between baseline and six months after TIPS correlated positively with changes in IL‐6 and negatively with changes in ammonia. This might indicate that a decrease in systemic inflammation seems to be associated with a decrease in NfL or GFAP, while ammonia seemed to be linked in an unexpected way. This finding also adds to a study by Fiorillo et al. demonstrating that Rifaximin treatment in patients with MHE is associated with a decrease of NfL levels [11]. According to our longitudinal analyses, the decrease of NfL serum levels seems to be independent of the toxic effects of ammonia and seems to be more dependent on decreasing systemic inflammation. In this context, we found a significant decrease in IL‐6 and CRP levels between baseline and months six, while we saw a 35% increase in ammonia levels. This is well in line with other studies demonstrating a decrease in systemic inflammation after TIPS insertion [14]. These aforementioned longitudinal and prospective findings add to the evidence of a cross‐sectional study published by our group, by validating the robust correlation between systemic inflammation and NfL serum levels in patients with cirrhosis [9]. The plausibility of a robust correlation between NfL serum levels and systemic inflammation can also be found in studies investigating the biomarker in the context of other diseases. In this context, most evidence is available for multiple sclerosis, a chronic inflammatory disease of the central nervous system [15, 16, 17].

Our findings have to be interpreted considering the contextual factors of our cohort. The predominant indication for TIPS implantation was ascites with 72% of the cases. These patients usually have a poorer prognosis compared to patients with TIPS after failure of secondary prophylaxis after variceal bleeding. This also explains the comparably high OHE rate in our study. However, our findings might not apply to other indications for TIPS insertion, such as an early TIPS for variceal bleeding, and certainly need to be validated in other patient cohorts.

Our study has limitations that have to be acknowledged. The case numbers for the longitudinal part of the study investigating the trajectory of NfL and GFAP levels are comparatively small and not all patients could be sampled at all time points. In addition, this analysis is also prone to selection bias towards healthier patients, because patients had to be alive without liver transplantation and had to attend follow‐up visits. Therefore, these results should be interpreted with caution. Another limitation is that the subgroup of patients with a TIPS after failure of secondary prophylaxis after variceal bleeding is comparably small precluding further analyses in this group. Therefore, our findings need additional validation in a larger cohort of patients with TIPS after variceal bleeding. Given that patients with a TIPS for variceal bleeding differ from patients with a TIPS for refractory ascites, it might be possible that our findings do not fully apply to this group. The same applies for subgroup analyses of patients stratified according to the underlying etiology of liver disease. This may be especially interesting in patients with ALD, as alcohol‐related peripheral neuropathy may influence circulating NfL levels [18]. However, we did not include patients with active alcohol consumption at TIPS insertion minimizing this potential bias. On the flipside, our study cannot answer the question whether NfL levels are also useful in patients with TIPS and ongoing alcohol consumption. Moreover, the group of patients with viral hepatitis as main etiology of cirrhosis is small. Therefore, our results cannot be generalized to cohorts from other continents, such as Asia, and need proper validation.

In conclusion, we provide insight in the longitudinal trajectory of NfL and GFAP serum levels after TIPS insertion. In addition and most importantly, we found that higher NfL levels in patients with cirrhosis before TIPS are associated with post‐TIPS OHE and transplantation‐free survival. NfL levels before TIPS insertion might allow a more granular risk stratification regarding HE risk and overall prognosis independent of other established prognostic variables.

## 4 Patient and Methods

### 4.1 Study Population

Patients with cirrhosis from three tertiary care centers in Germany were prospectively recruited: University Medical Center Mainz (2022–2024), Hannover Medical School (2019–2024), and University Medical Center Schleswig‐Holstein Campus Lübeck (2022–2023). Data analysis was done retrospectively. The study protocols for Mainz and Hannover were registered at clinicaltrials.gov (NCT05466669 and NCT04801290). Patients from Lübeck were recruited following the Mainz protocol. Only patients requiring elective TIPS implantation for refractory ascites, secondary prophylaxis after variceal hemorrhage or ascites and an additional portal vein thrombosis were included. Exclusion criteria were: Patients undergoing rescue or early TIPS implantation, no cirrhosis, unwillingness to participate, ongoing alcohol consumption at the time of TIPS placement, severe neurological comorbidities or chronic kidney disease requiring hemodialysis. These patients were not considered for this study [19].

Follow‐up examinations were performed one‐, three‐, and six‐months post‐TIPS in the outpatient departments of the study centers. Patients were then followed up every six months in line with recommendations for HCC surveillance. Cirrhosis was diagnosed by histology or a combination of blood chemistry, sonography, radiological imaging, and endoscopic findings.

### 4.2 TIPS Insertion

TIPS insertion was performed by the Departments of Diagnostic and Interventional Radiology at each center according to institutional standard operating procedures under general anesthesia. Only polytetrafluoroethylene‐covered stent grafts (GORE VIATORR TIPS Endoprothesis, Flagstaff, Arizona, AZ, USA) were inserted. Portosystemic gradient (PSG) measurements were done in line with the recommendations of the BAVENO VII consensus [20]. The TIPS diameter was adjusted to reach a PSG target of less than 12 mmHg. A second PSG measurement was done after 2–3 days under local anesthesia in patients from Mainz and Lübeck. If the second PSG was above 12 mmHg, then the TIPS was further dilated. The final stent diameters were: 6 mm (*n* = 33), 7 mm (*n* = 3), 8 mm (*n* = 74), and 10 mm (*n* = 34).

### 4.3 Determination of NfL and GFAP Serum Levels

Both NfL and GFAP serum levels were determined using the highly sensitive single‐molecule array (SiMoA) technology as described elsewhere [9, 10]. The measurement of all samples was carried out centrally in Mainz. Measurements were performed in a blinded fashion without information about clinical data.

### 4.4 Determination of Ammonia and Interleukin‐6 Serum Levels

Ammonia levels were measured in patients from Mainz and Hannover following a predefined standard operating procedure. Blood samples were transported on ice to the central laboratory within five minutes and measured within 20–30 min. Ammonia levels were normalized to the upper limit of normal (Mainz: 72 µmol/L, Hannover: 60 µmol/L). In Mainz, Interleukin‐6 (IL‐6) blood levels were determined with a commercially available chemiluminescence immunoassay (Cobas e 411 Analyzer, F. Hoffmann‐La Roche AG, Basel, Swiss).

### 4.5 Diagnosis of HE

The portosystemic encephalopathy (PSE) syndrome test was used to test for CHE within two weeks before TIPS implantation. Interpretation of PHES was done as previously described with German norms (version 2.0; 2020) [21, 22]. A PHES lower than −4 was considered pathological.

### 4.6 Follow‐Up Evaluations

Patients were followed for the development of overt hepatic encephalopathy (overt HE, OHE), liver transplantation and/or death after TIPS insertion. During each outpatient visit and unplanned hospitalization, an experienced hepatologist examined each patient to rule in or rule out OHE. OHE was diagnosed following a complete neurological examination in accordance with the West‐Haven criteria. In the case of external hospitalizations, data were obtained from the respective hospitals. For secondary analyses for the evaluation of longitudinal changes in NfL and GFAP after TIPS insertion, patients from Mainz were examined at predefined visits after one, three, and six months after TIPS insertion during outpatient appointments. Here, blood samples were stored for measurement of NfL and GFAP levels at baseline and after months one and six. Samples were not available for all patients because competing events (death or liver transplantation) occurred, patients did not appear at the predefined follow‐up visit, or the follow‐up period of six months had not been reached.

### 4.7 Ethics

The studies were approved by the ethics committees of Hannover Medical School (Nr. 8498_BO_S_2019), the Landesärztekammer Rheinland‐Pfalz (Nr. 2021–16247_1) and Lübeck (Nr. 21–448). All patients provided written informed consent. The study was conducted in accordance with principles of the Declaration of Helsinki.

### 4.8 Statistical Analysis

Data analysis was carried out with R 4.3.3 (R Core Team (2024). R: A Language and Environment for Statistical Computing. R Foundation for Statistical Computing, Vienna, Austria. https://www.R‐project.org/.), RStudio version 2023.12.1.402 (Posit team (2024). RStudio: Integrated Development Environment for R. Posit Software, PBC, Boston, MA. URL http://www.posit.co/), IBM SPSS Statistic Version 29.0 (Armonk, NY: IBM Corp.) and GraphPad Prism Version 8.0.2 (GraphPad Software, California, US). Categorical variables are presented as numbers with percentages, continuous cariables as median with interquartile range (IQR). Pairwise comparisons were assessed with the Wilcoxon rank sum test, Pearson`s Chi‐squared test or Fisher`s exact test, as appropriate. Comparisons between paired groups were done with Friedman's test. For correlation analyses, Spearman`s rank correlation coefficient was used.

The {tidycmprsk} R package (v0.2.0, Daniel D. Sjoberg and Teng Fei 2022) was used for (i) cumulative incidence functions for competing risk analyses and (ii) Fine and Gray competing risk regression analyses. Multi‐state models with three states were implemented to analyze OHE development (0: alive, no liver transplantation, no OHE event at the end of follow‐up, 1: OHE event during follow‐up, 2: death or liver transplantation without prior OHE event during follow‐up).

For liver transplantation‐free survival analysis, Kaplan–Meier curves and Cox proportional hazards regression models were fitted. Group differences in Kaplan–Meier curve analysis was assessed with the log‐rank test. In all Cox analyses, a two‐state model with death and liver transplantation as composite endpoints was used (0: alive, not transplanted; 1: dead or liver transplanted).

To investigate non‐linear effects of NfL or GFAP on OHE development, NfL and GFAP serum concentrations were fitted using restricted cubic splines with four knots in a Cox model ({rms}TR package, v6.8‐0, Harrell Jr FE, 2024). A complete case analysis was made in case of missing data.

## Author Contributions

Performed research: All authors. Contributed to acquisition of data: All authors. Designed the study and analyzed the data: CL. Statistical analysis: CL. Wrote the paper: CL. Critical revision of the manuscript: All authors. All authors have read and approved the final manuscript. Guarantor of the article: Christian Labenz

## Funding

This work was partly supported by the German Center for Infectious Research (no approval number available) and by the Dr. Rolf M. Schwiete Stiftung (grant number: 2022–56).

## Ethics Statement

The studies were approved by the ethics committees of Hannover Medical School (Nr. 8498_BO_S_2019), the Landesärztekammer Rheinland‐Pfalz (Nr. 2021–16247_1) and Lübeck (Nr. 21–448). All patients provided written informed consent. The study was conducted in accordance with principles of the Declaration of Helsinki. Registration numbers: NCT05466669 and NCT04801290.

## Conflicts of Interest

CL: Lecture and consultant fees: Merz Therapeutics, Norgine, Intercept, Gilead Sciences, Ipsen, Falk Foundation e.V., CSL Behring, Boehringer Ingelheim. Research grants: Merz Therapeutics, Norgine, Schwiete Foundation.

EMS: received travel grants from Abbvie and Advitos.

MM: received consultant fees from AstraZeneca.

PRG: Lecture fees and consulting: Merz Pharmaceuticals

SB: S.B. has received honoraria from Biogen, Bristol Myers Squibb, Hexal, Merck Healthcare, Novartis, Roche, Sanofi and Teva. S.B. is supported by the Deutsche Forschungsgemeinschaft (DFG, SFB CRC TRR 355 ‐ 480846870), Novartis and the Hermann‐ and Lilly‐Schilling Foundation.

BM: Lecture and consultant fees: AstraZeneca, AbbVie, Fujirebio, Falk Foundation e.V., Gilead, Luvos, MSD, Norgine, Roche, W. L. Gore & Associates. Research grants: Altona, EWIMED, Fujirebio and Roche.

JUM: Lecture and consultant fees: Merz Therapeutics, Falk Foundation e.V., ABBVIE, AstraZeneca, Gilead; Research grants: Merz Therapeutics

SJG: Travel expenses: Ipsen and Gilead. Research grant: Schwiete Foundation.

All other authors disclose no potential financial or non‐financial conflict of interests regarding this study.

## Supporting information




**Supporting Figure S1**: Patient flow‐chart.
**Supporting Figure S2**: Density plot of OHE events post‐TIPS.
**Supporting Figure S3**: Non‐linear effect of NfL (A) and GFAP (B) serum levels on post‐TIPS HE risk. An univariable Cox model with NfL or GFAP as restricted cubic spline with four knots was fitted.
**Supporting Figure S4**: Longitudinal changes of NfL and GFAP serum levels after TIPS insertion.
**Supporting Figure 1A**: displays the trajectory of NfL serum levels after TIPS insertion at 30 and 180 days (n = 16 at all time points). Fig. 1B displays the trajectory of GFAP serum levels after TIPS insertion at 30 and 180 days (n = 16 at all time points). Fig. 1C and 1D display the individual trajectories of NfL (C) and GFAP (D) serum levels 30 days after TIPS insertion (n = 35). Fig. 1E and 1F display the individual trajectories of NfL (E) and GFAP (F) serum levels 180 days after TIPS insertion (n = 21). **p < 0.01.
**Supporting Figure S5**: Comparison of delta NfL (A) and delta GFAP (B) serum levels between patients with or without a post‐TIPS HE.
The deltas display NfL or GFAP levels at 180 days minus levels at baseline prior to TIPS. N = 21
**Supporting Figure S6**: Correlation analyses of deltas (180 days—baseline) of different variables.
The deltas display the values of the respective variables at 180 days minus the values at baseline prior to TIPS. Spearman's rank correlation. N = 21
**Supporting Table S1**: Comparisons of demographics and characteristics of the study cohort stratified by NfL median.
**Supporting Table S2**: Comparisons of demographics and characteristics of the study cohort stratified by GFAP median.
**Supporting Table S3**: Multivariable Fine and Gray regression analysis for OHE development.
**Supporting Table S4**: Multivariable Fine and Gray regression analysis for OHE development.
**Supporting Table S5**: Multivariable Fine and Gray regression analysis for OHE development.
**Supporting Table S6**: Multivariable Fine and Gray regression analysis for OHE development.
**Supporting Table S7**: Multivariable Fine and Gray regression analysis for OHE development in the subgroup of patients without a history of OHE prior to TIPS.
**Supporting Table S8**: Multivariable Fine and Gray regression analysis for OHE development in the subgroup of patients with ascites as TIPS indication.
**Supporting Table S9**: Multivariable Fine and Gray regression analysis for OHE development in the subgroup of patients with ascites as TIPS indication.
**Supporting Table S10**: Univariable Cox regression analysis for death/liver transplantation.

## Data Availability

Raw data are available from the corresponding author on reasonable request.
