# Supplementary material for: Neurofilament Light Chains in Serum Predict Post—Transjugular Intrahepatic Portosystemic Shunt Hepatic Encephalopathy
Source: MedComm (2020). 2025 Nov 5;6(11):e70475. doi: 10.1002/mco2.70475 (PMC12587166; doi:10.1002/mco2.70475)
Supplement: Supplementary file 1 — Supporting Figure S1: Patient flow‐chart. Supporting Figure S2: Density plot of OHE events post‐TIPS. Supporting Figure S3: Non‐linear effect of NfL (A) and GFAP (B) serum levels on post‐TIPS HE risk. An univariable Cox model with NfL or GFAP as restricted cubic spline with four knots was fitted. Supporting Figure S4: Longitudinal changes of NfL and GFAP serum levels after TIPS insertion. Supporting Figure 1A: displays the trajectory of NfL serum levels after TIPS insertion at 30 and 180 days (n = 16 at all time points). Fig. 1B displays the trajectory of GFAP serum levels after TIPS insertion at 30 and 180 days (n = 16 at all time points). Fig. 1C and 1D display the individual trajectories of NfL (C) and GFAP (D) serum levels 30 days after TIPS insertion (n = 35). Fig. 1E and 1F display the individual trajectories of NfL (E) and GFAP (F) serum levels 180 days after TIPS insertion (n = 21). **p < 0.01. Supporting Figure S5: Comparison of delta NfL (A) and delta GFAP (B) serum levels between patients with or without a post‐TIPS HE. The deltas display NfL or GFAP levels at 180 days minus levels at baseline prior to TIPS. N = 21 Supporting Figure S6: Correlation analyses of deltas (180 days—baseline) of different variables. The deltas display the values of the respective variables at 180 days minus the values at baseline prior to TIPS. Spearman's rank correlation. N = 21 Supporting Table S1: Comparisons of demographics and characteristics of the study cohort stratified by NfL median. Supporting Table S2: Comparisons of demographics and characteristics of the study cohort stratified by GFAP median. Supporting Table S3: Multivariable Fine and Gray regression analysis for OHE development. Supporting Table S4: Multivariable Fine and Gray regression analysis for OHE development. Supporting Table S5: Multivariable Fine and Gray regression analysis for OHE development. Supporting Table S6: Multivariable Fine and Gray regression analysis for OHE development. Supporting Table S [file MCO2-6-e70475-s001.docx]

**Neurofilament light chains in serum predict post-TIPS hepatic encephalopathy**

Christian Labenz^1,2#^, Eva Maria Schleicher^1,2^, Myriam Meineck^1^, Martin Kabelitz^3^, Alena Ehrenbauer^3^, Anja Tiede^3^, Jim Mauz^3^, Sven Danneberg^4^, Michael Bernhard Pitton^5^, Falk Steffen^6^, Julia Weinmann-Menke^1^, Peter Robert Galle^1,2^, Stefan Bittner^6^, Felix Lüssi^6^, Jens Uwe Marquardt^4^*, Benjamin Maasoumy^3^*, Simon Johannes Gairing^1,2^*

**Table of contents**

[Fig. S1. Patient flow-chart. 2](#_Toc191990906)

[Fig. S2. Density plot of OHE events post-TIPS. 3](#_Toc191990907)

[Fig. S3. Non-linear effect of NfL (A) and GFAP (B) serum levels on post-TIPS HE risk. 4](#_Toc191990908)

[Fig. S4. Longitudinal changes of NfL and GFAP serum levels after TIPS insertion. 5](#_Toc191990909)

[Fig. S5. Comparison of delta NfL (A) and delta GFAP (B) serum levels between patients with or without a post-TIPS HE. 6](#_Toc191990910)

[Fig. S6. Correlation analyses of deltas (180 days – baseline) of different variables. 7](#_Toc191990911)

[Table S1: Comparisons of demographics and characteristics of the study cohort stratified by NfL median. 8](#_Toc191990912)

[Table S2: Comparisons of demographics and characteristics of the study cohort stratified by GFAP median. 10](#_Toc191990913)

[Table S3: Multivariable Fine and Gray regression analysis for OHE development. 12](#_Toc191990914)

[Table S4: Multivariable Fine and Gray regression analysis for OHE development. 13](#_Toc191990915)

[Table S5: Multivariable Fine and Gray regression analysis for OHE development. 14](#_Toc191990916)

[Table S6: Multivariable Fine and Gray regression analysis for OHE development. 15](#_Toc191990917)

[Table S7: Multivariable Fine and Gray regression analysis for OHE development in the subgroup of patients without a history of OHE prior to TIPS. 16](#_Toc191990918)

[Table S8: Multivariable Fine and Gray regression analysis for OHE development in the subgroup of patients with ascites as TIPS indication. 17](#_Toc191990919)

[Table S9: Multivariable Fine and Gray regression analysis for OHE development in the subgroup of patients with ascites as TIPS indication. 18](#_Toc191990920)

[Table S10: Univariable Cox regression analysis for death/liver transplantation. 19](#_Toc191990921)

**
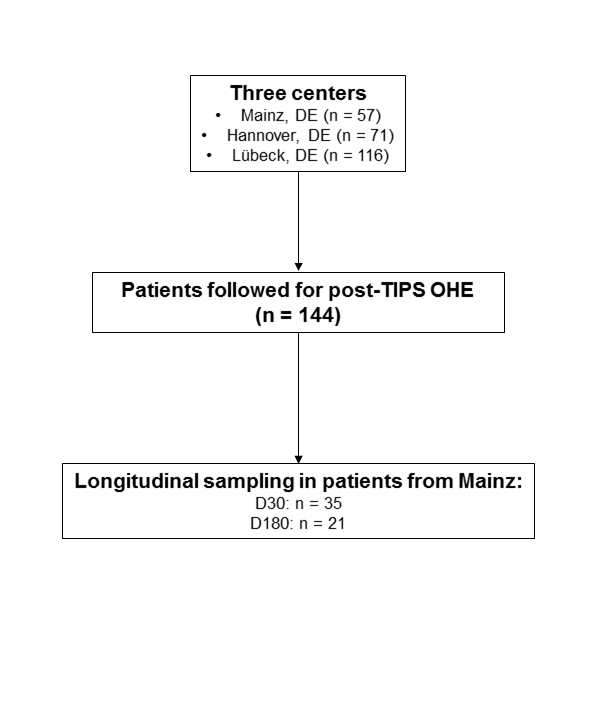
**

# Fig. S1. Patient flow-chart.


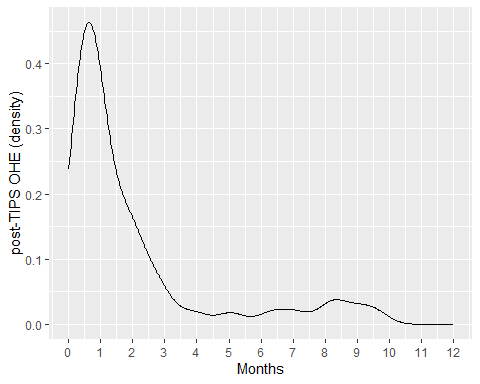


# Fig. S2. Density plot of OHE events post-TIPS.

**(A)**


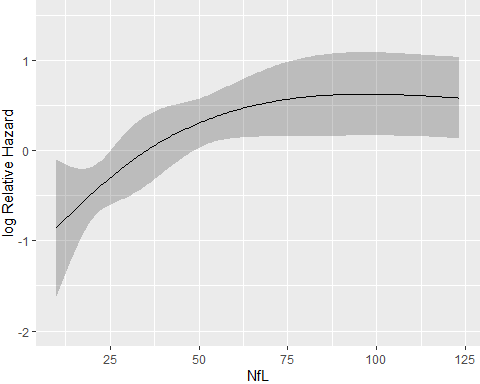


**(B)**


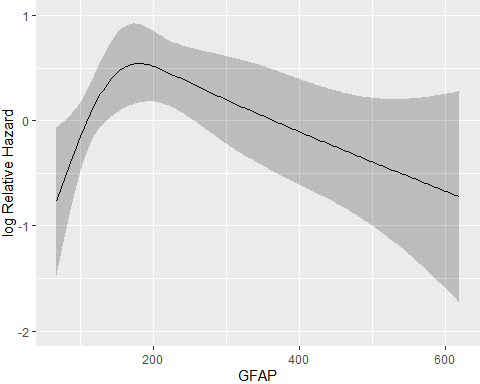


# Fig. S3. Non-linear effect of NfL (A) and GFAP (B) serum levels on post-TIPS HE risk.

An univariable Cox model with NfL or GFAP as restricted cubic spline with four knots was fitted.


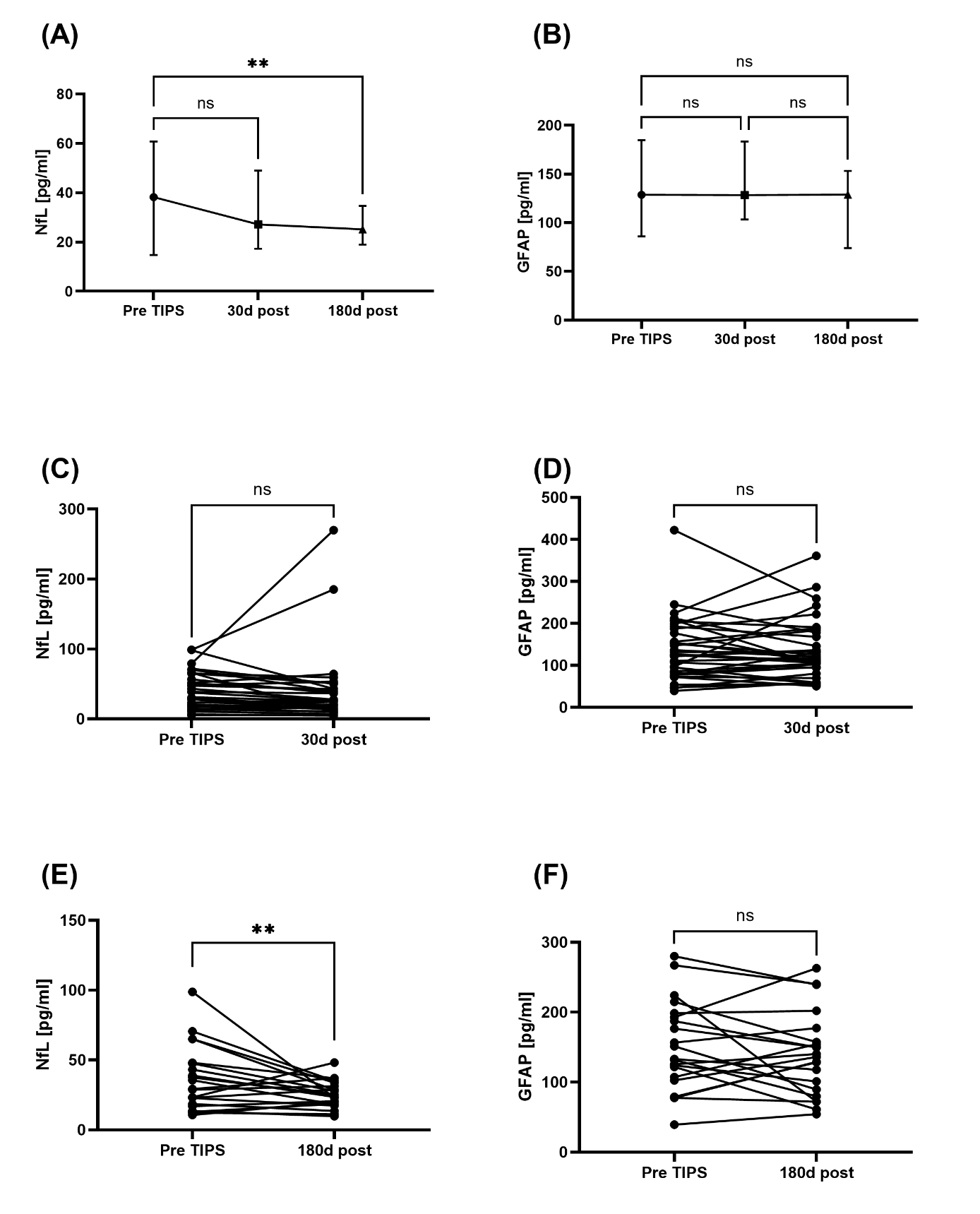


# Fig. S4. Longitudinal changes of NfL and GFAP serum levels after TIPS insertion.

Fig. 1A displays the trajectory of NfL serum levels after TIPS insertion at 30 and 180 days (n = 16 at all time points). Fig. 1B displays the trajectory of GFAP serum levels after TIPS insertion at 30 and 180 days (n=16 at all time points). Fig. 1C and 1D display the individual trajectories of NfL (C) and GFAP (D) serum levels 30 days after TIPS insertion (n = 35). Fig. 1E and 1F display the individual trajectories of NfL (E) and GFAP (F) serum levels 180 days after TIPS insertion (n = 21). **p < 0.01.

**
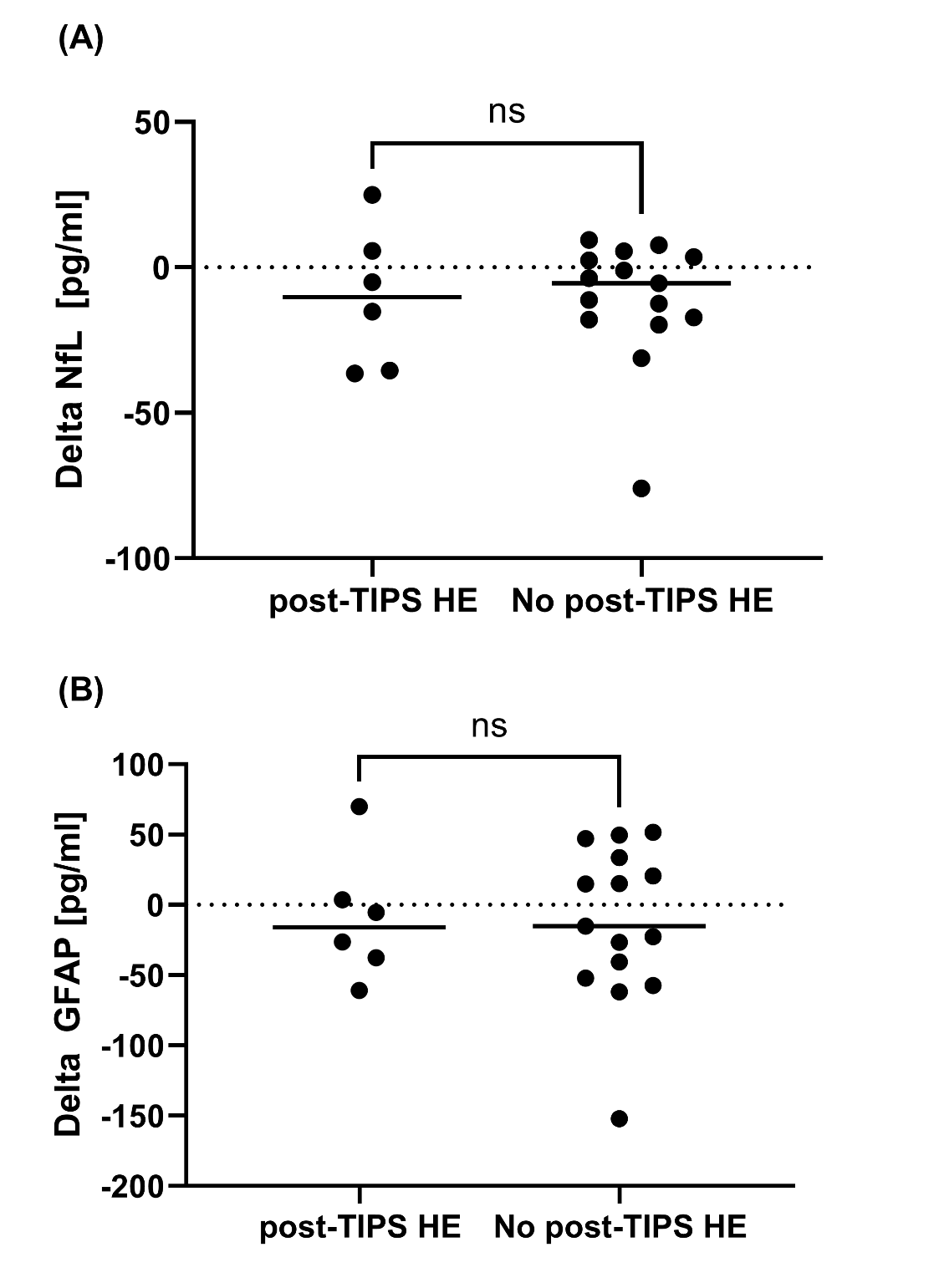
**

# Fig. S5. Comparison of delta NfL (A) and delta GFAP (B) serum levels between patients with or without a post-TIPS HE.

The deltas display NfL or GFAP levels at 180 days minus levels at baseline prior to TIPS. N = 21


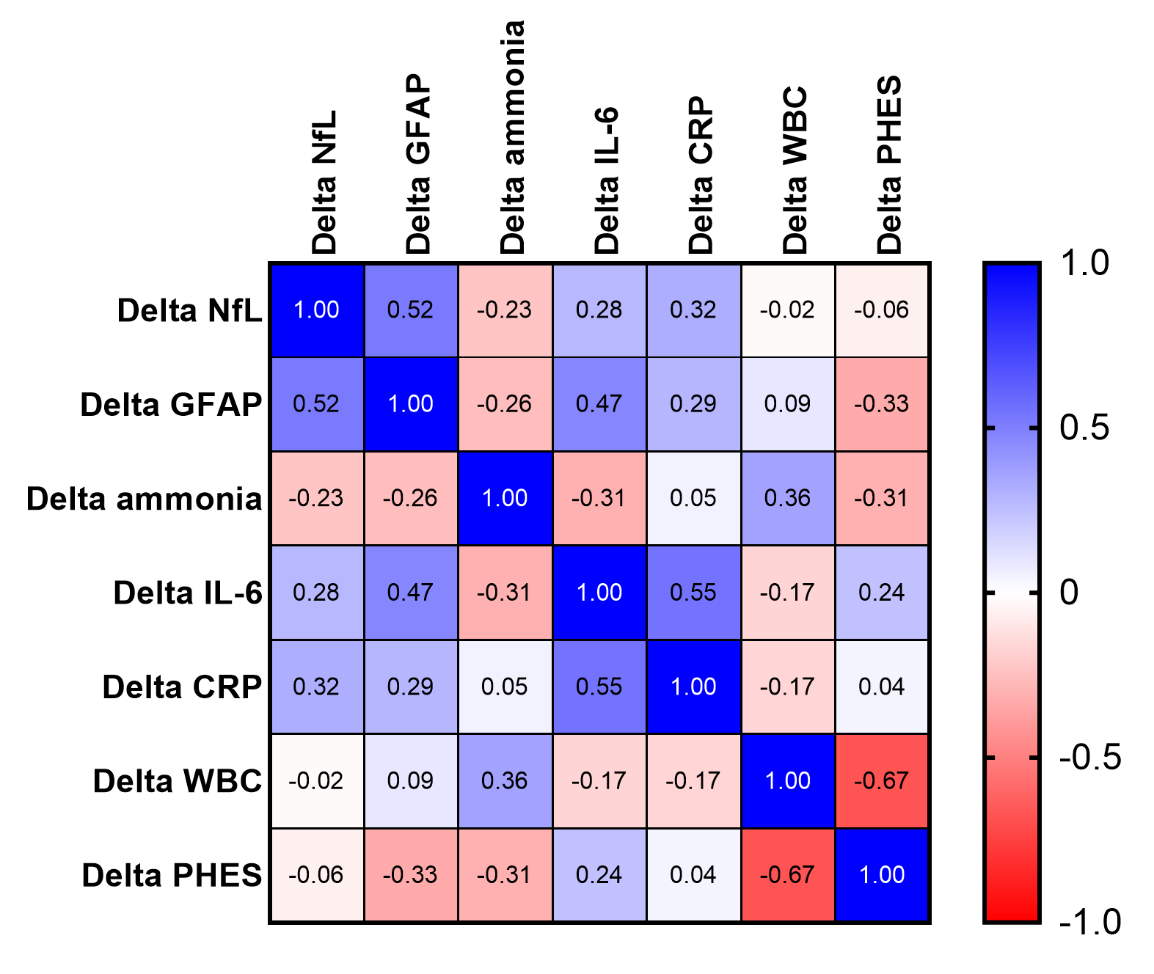


# Fig. S6. Correlation analyses of deltas (180 days – baseline) of different variables.

The deltas display the values of the respective variables at 180 days minus the values at baseline prior to TIPS. Spearman’s rank correlation. N = 21

# Table S1: Comparisons of demographics and characteristics of the study cohort stratified by NfL median.

| **Variable** | **N** | **NfL < median**  N = 72 | **NfL ≥ median**  N = 72 | **p-value***^1^* |
| --- | --- | --- | --- | --- |
| **Age (years)** | 144 | 60 (52, 65) | 63 (53, 69) | 0.13 |
| **Sex** | 144 |  |  | 0.2 |
| male |  | 53 (74%) | 46 (64%) |  |
| female |  | 19 (26%) | 26 (36%) |  |
| **Indication for TIPS** | 144 |  |  | 0.3 |
| Ascites |  | 48 (67%) | 55 (76%) |  |
| Bleeding |  | 19 (26%) | 15 (21%) |  |
| Other |  | 5 (6.9%) | 2 (2.8%) | 0.1 |
| **Etiology** | 144 |  |  | 0.1 |
| ALD |  | 33 (46%) | 37 (51%) |  |
| MetALD |  | 1 (1.4%) | 7 (9.7%) |  |
| MASLD |  | 14 (19%) | 6 (8.3%) |  |
| Viral |  | 5 (6.9%) | 3 (4.2%) |  |
| Other |  | 14 (19%) | 16 (22%) |  |
| ALD+Viral |  | 5 (6.9%) | 3 (4.2%) |  |
| **PSG Delta (%)** | 144 | 60 (50, 70) | 57 (47, 66) | 0.4 |
| **TIPS diameter (mm)** | 144 |  |  | 0.4 |
| 6 |  | 13 (18%) | 20 (28%) |  |
| 7 |  | 2 (2.8%) | 1 (1.4%) |  |
| 8 |  | 37 (51%) | 37 (51%) |  |
| 10 |  | 20 (28%) | 14 (19%) |  |
| **CHE (PHES)** | 124 |  |  | **<0.001** |
| CHE- |  | 41 (65%) | 11 (18%) |  |
| CHE+ |  | 22 (35%) | 50 (82%) |  |
| **PHES** | 123 | -3.0 (-6.0, -1.0) | -7.0 (-10.0, -5.0) | **<0.001** |
| **Child Pugh** | 144 |  |  | 0.5 |
| A |  | 13 (18%) | 8 (11%) |  |
| B |  | 51 (71%) | 57 (79%) |  |
| C |  | 8 (11%) | 7 (9.7%) |  |
| **FIPS score** | 144 | -0.35 (-0.81, 0.30) | -0.02 (-0.58, 0.45) | 0.065 |
| **MELD score** | 144 | 11.3 (9.1, 14.0) | 12.1 (9.1, 14.6) | 0.4 |
| **History of OHE** | 144 | 10 (14%) | 30 (42%) | **<0.001** |
| **Sodium (mmol/L)** | 142 | 136.0 (134.0, 138.0) | 135.0 (133.0, 138.0) | 0.14 |
| **Creatinine (mg/dL)** | 144 | 0.99 (0.85, 1.30) | 1.19 (0.93, 1.75) | **0.007** |
| **Bilirubin (mg/dL)** | 144 | 1.11 (0.81, 1.90) | 1.03 (0.59, 1.43) | **0.043** |
| **Albumin (g/L)** | 144 | 34.0 (29.5, 38.0) | 29.0 (26.0, 34.0) | **<0.001** |
| **CRP (mg/L)** | 142 | 7 (3, 15) | 10 (5, 23) | 0.059 |
| **WBC (per nL)** | 143 | 5.10 (3.65, 7.80) | 5.08 (3.70, 7.18) | >0.9 |
| **Hemoglobin (g/dL)** | 143 | 10.85 (9.60, 12.25) | 9.30 (8.10, 10.50) | **<0.001** |
| **Platelets (per nL)** | 142 | 108 (63, 166) | 112 (75, 181) | 0.4 |
| **Ammonia pre-TIPS (ULN)** | 126 | 0.71 (0.58, 0.86) | 0.76 (0.52, 1.06) | 0.6 |
| **NfL (pg/ml)** | 144 | 23 (15, 29) | 65 (51, 94) | **<0.001** |
| **GFAP (pg/ml)** | 144 | 133 (82, 201) | 237 (177, 351) | **<0.001** |
| **Lactulose** | 144 | 41 (57%) | 41 (57%) | 0.9 |
| **Rifaximin** | 144 | 31 (43%) | 38 (53%) | 0.2 |
| *^1^*Wilcoxon rank sum test, Pearson's Chi-squared test or Fisher's exact test, as appropriate. | | | | |

Data are expressed as median with interquartile range or as number with percentages. Abbr.: CHE, covert hepatic encephalopathy; PHES, psychometric hepatic encephalopathy score; NfL, neurofilament light chains; GFAP, glial fibrillary acidic protein; PSG, portosystemic gradient; MELD, model for end-stage liver disease; OHE, overt hepatic encephalopathy.

# Table S2: Comparisons of demographics and characteristics of the study cohort stratified by GFAP median.

| **Variable** | **N** | **GFAP < median**  N = 72 | **GFAP ≥ median**  N = 72 | **p-value***^1^* |
| --- | --- | --- | --- | --- |
| **Age (years)** | 144 | 56 (50, 64) | 63 (57, 70) | **0.001** |
| **Sex** | 144 |  |  | **0.007** |
| male |  | 57 (79%) | 42 (58%) |  |
| female |  | 15 (21%) | 30 (42%) |  |
| **Indication for TIPS** | 144 |  |  | >0.9 |
| Ascites |  | 52 (72%) | 51 (71%) |  |
| Bleeding |  | 17 (24%) | 17 (24%) |  |
| Other |  | 3 (4.2%) | 4 (5.6%) |  |
| **Etiology** | 144 |  |  | 0.4 |
| ALD |  | 36 (50%) | 34 (47%) |  |
| MetALD |  | 2 (2.8%) | 6 (8.3%) |  |
| MASLD |  | 10 (14%) | 10 (14%) |  |
| Viral |  | 6 (8.3%) | 2 (2.8%) |  |
| Other |  | 13 (18%) | 17 (24%) |  |
| ALD+Viral |  | 5 (6.9%) | 3 (4.2%) |  |
| **PSG Delta (%)** | 144 | 59 (50, 67) | 58 (48, 69) | 0.9 |
| **TIPS diameter (mm)** | 144 |  |  | 0.2 |
| 6 |  | 14 (19%) | 19 (26%) |  |
| 7 |  | 1 (1.4%) | 2 (2.8%) |  |
| 8 |  | 35 (49%) | 39 (54%) |  |
| 10 |  | 22 (31%) | 12 (17%) |  |
| **CHE (PHES)** | 124 |  |  | **<0.001** |
| CHE- |  | 39 (63%) | 13 (21%) |  |
| CHE+ |  | 23 (37%) | 49 (79%) |  |
| **PHES** | 123 | -3.0 (-6.0, -1.0) | -7.0 (-9.0, -5.0) | **<0.001** |
| **Child Pugh** | 144 |  |  | 0.7 |
| A |  | 10 (14%) | 11 (15%) |  |
| B |  | 53 (74%) | 55 (76%) |  |
| C |  | 9 (13%) | 6 (8.3%) |  |
| **FIPS score** | 144 | -0.29 (-0.83, 0.35) | -0.16 (-0.54, 0.45) | 0.14 |
| **MELD score** | 144 | 12.0 (9.3, 14.0) | 11.0 (9.0, 14.9) | 0.8 |
| **History of OHE** | 144 | 14 (19%) | 26 (36%) | **0.026** |
| **Sodium (mmol/L)** | 142 | 136.0 (134.0, 138.0) | 135.0 (132.0, 138.0) | 0.2 |
| **Creatinine (mg/dL)** | 144 | 0.99 (0.85, 1.38) | 1.18 (0.94, 1.68) | **0.021** |
| **Bilirubin (mg/dL)** | 144 | 1.23 (0.84, 1.87) | 0.92 (0.59, 1.50) | **0.024** |
| **Albumin (g/L)** | 144 | 33.5 (29.0, 37.3) | 29.5 (26.0, 35.0) | **0.004** |
| **CRP (mg/L)** | 142 | 8 (4, 17) | 9 (4, 16) | 0.5 |
| **WBC (per nL)** | 143 | 4.60 (3.60, 7.00) | 5.18 (3.80, 7.80) | 0.2 |
| **Hemoglobin (g/dL)** | 143 | 10.70 (9.10, 12.10) | 9.60 (8.60, 10.85) | **0.014** |
| **Platelets (per nL)** | 142 | 112 (64, 172) | 110 (74, 181) | 0.6 |
| **Ammonia pre-TIPS (ULN)** | 126 | 0.72 (0.57, 0.94) | 0.72 (0.56, 0.88) | 0.6 |
| **NfL (pg/ml)** | 144 | 25 (16, 42) | 56 (34, 87) | **<0.001** |
| **GFAP (pg/ml)** | 144 | 117 (79, 151) | 298 (218, 373) | **<0.001** |
| **Lactulose** | 144 | 39 (54%) | 43 (60%) | 0.5 |
| **Rifaximin** | 144 | 30 (42%) | 39 (54%) | 0.1 |
| *^1^*Wilcoxon rank sum test, Pearson's Chi-squared test or Fisher's exact test, as appropriate. | | | | |
|  | | | | |

Data are expressed as median with interquartile range or as number with percentages. Abbr.: CHE, covert hepatic encephalopathy; PHES, psychometric hepatic encephalopathy score; NfL, neurofilament light chains; GFAP, glial fibrillary acidic protein; PSG, portosystemic gradient; MELD, model for end-stage liver disease; OHE, overt hepatic encephalopathy.

# Table S3: Multivariable Fine and Gray regression analysis for OHE development.

| **Characteristic** | **N** | **sHR** | **95% CI** | **p-value** |
| --- | --- | --- | --- | --- |
| NfL (pg/ml) | 142 | 1.01 | 1.00, 1.01 | 0.028 |
| PSG delta (%) | 142 | 1.00 | 0.98, 1.02 | 0.9 |
| MELD | 142 | 1.05 | 0.98, 1.12 | 0.2 |
| History of OHE |  |  |  |  |
| 0 | 102 | — | — |  |
| 1 | 40 | 1.15 | 0.62, 2.12 | 0.7 |
| Platelets | 142 | 1.00 | 0.99, 1.00 | 0.028 |
| Age | 142 | 1.02 | 0.99, 1.04 | 0.2 |
| Lactulose |  |  |  |  |
| 0 | 61 | — | — |  |
| 1 | 81 | 0.97 | 0.55, 1.71 | >0.9 |
| Rifaximin |  |  |  |  |
| 0 | 74 | — | — |  |
| 1 | 68 | 0.88 | 0.51, 1.53 | 0.7 |
|  | | | | |

In multivariable Fine and Gray regression analysis for OHE development, liver transplantation and death were treated as competing events. Abbr.: sHR, subdistribution hazard ratio; CI, confidence interval; NfL, neurofilament light chains; PSG, portosystemic gradient; MELD, model for end-stage liver disease; OHE, overt hepatic encephalopathy.

# Table S4: Multivariable Fine and Gray regression analysis for OHE development.

| **Characteristic** | **N** | **sHR** | **95% CI** | **p-value** |
| --- | --- | --- | --- | --- |
| GFAP (pg/ml) | 142 | 1.00 | 1.00, 1.00 | 0.2 |
| PSG delta (%) | 142 | 1.00 | 0.98, 1.02 | 0.8 |
| MELD | 142 | 1.04 | 0.98, 1.12 | 0.2 |
| History of OHE |  |  |  |  |
| 0 | 102 | — | — |  |
| 1 | 40 | 1.35 | 0.73, 2.51 | 0.3 |
| Age | 142 | 1.03 | 1.00, 1.05 | 0.060 |
| Platelets | 142 | 1.00 | 0.99, 1.00 | 0.11 |
| Lactulose |  |  |  |  |
| 0 | 61 | — | — |  |
| 1 | 81 | 0.89 | 0.50, 1.57 | 0.7 |
| Rifaximin |  |  |  |  |
| 0 | 74 | — | — |  |
| 1 | 68 | 1.00 | 0.57, 1.75 | >0.9 |
|  | | | | |

In multivariable Fine and Gray regression for OHE development, liver transplantation and death were treated as a competing event. Abbr.: sHR, subdistribution hazard ratio; CI, confidence interval; NfL, neurofilament light chains; PSG, portosystemic gradient; MELD, model for end-stage liver disease; OHE, overt hepatic encephalopathy.

# Table S5: Multivariable Fine and Gray regression analysis for OHE development.

| **Characteristic** | **N** | **sHR** | **95% CI** | **p-value** |
| --- | --- | --- | --- | --- |
| NfL (pg/mL) | 142 | 1.01 | 1.00, 1.01 | 0.021 |
| PSG delta (%) | 142 | 1.00 | 0.98, 1.02 | 0.9 |
| FIPS | 142 | 1.34 | 0.93, 1.94 | 0.12 |
| History of OHE |  |  |  |  |
| 0 | 102 | — | — |  |
| 1 | 40 | 1.17 | 0.64, 2.15 | 0.6 |
| Platelets (/nl) | 142 | 1.00 | 0.99, 1.00 | 0.014 |
|  | | | | |

In multivariable Fine and Gray regression for OHE development, liver transplantation and death were treated as a competing event. Abbr.: sHR, subdistribution hazard ratio; CI, confidence interval; NfL, neurofilament light chains; PSG, portosystemic gradient; MELD, model for end-stage liver disease; OHE, overt hepatic encephalopathy.

# Table S6: Multivariable Fine and Gray regression analysis for OHE development.

| **Characteristic** | **N** | **sHR** | **95% CI** | **p-value** |
| --- | --- | --- | --- | --- |
| NfL (pg/mL) | 142 | 1.01 | 1.00, 1.01 | 0.019 |
| PSG delta (%) | 142 | 1.00 | 0.98, 1.02 | >0.9 |
| Creatinine (mg/dL) | 142 | 1.44 | 1.17, 1.76 | <0.001 |
| Bilirubin (mg/dL) | 142 | 0.93 | 0.67, 1.29 | 0.7 |
| History of OHE |  |  |  |  |
| 0 | 102 | — | — |  |
| 1 | 40 | 1.03 | 0.54, 1.94 | >0.9 |
| Platelets (/nl) | 142 | 1.0 | 0.99, 1.00 | 0.009 |
| Age | 142 | 1.01 | 0.99, 1.04 | 0.3 |
|  | | | | |

In multivariable Fine and Gray regression for OHE development, liver transplantation and death were treated as a competing event. Abbr.: sHR, subdistribution hazard ratio; CI, confidence interval; NfL, neurofilament light chains; PSG, portosystemic gradient; MELD, model for end-stage liver disease; OHE, overt hepatic encephalopathy.

# Table S7: Multivariable Fine and Gray regression analysis for OHE development in the subgroup of patients without a history of OHE prior to TIPS.

| **Characteristic** | **N** | **sHR** | **95% CI** | **p-value** |
| --- | --- | --- | --- | --- |
| NfL (pg/mL) | 102 | 1.01 | 1.00, 1.01 | 0.005 |
| PSG delta (%) | 102 | 0.99 | 0.97, 1.01 | 0.3 |
| MELD | 102 | 1.02 | 0.94, 1.10 | 0.7 |
| Platelets (/nl) | 102 | 1.00 | 0.99, 1.00 | 0.093 |
| Age | 102 | 1.02 | 0.99, 1.06 | 0.2 |
|  | | | | |

In multivariable Fine and Gray regression for OHE development, liver transplantation and death were treated as a competing event. Abbr.: sHR, subdistribution hazard ratio; CI, confidence interval; NfL, neurofilament light chains; PSG, portosystemic gradient; MELD, model for end-stage liver disease; OHE, overt hepatic encephalopathy.

# Table S8: Multivariable Fine and Gray regression analysis for OHE development in the subgroup of patients with ascites as TIPS indication.

| **Characteristic** | **N** | **sHR** | **95% CI** | **p-value** |
| --- | --- | --- | --- | --- |
| NfL (pg/mL) | 102 | 1.01 | 1.00, 1.01 | <0.001 |
| PSG delta (%) | 102 | 1.01 | 0.99, 1.03 | 0.3 |
| MELD | 102 | 1.07 | 1.01, 1.15 | 0.034 |
| History of OHE |  |  |  |  |
| 0 | 73 | — | — |  |
| 1 | 29 | 1.79 | 0.95, 3.37 | 0.071 |
| Platelets (/nl) | 102 | 1.00 | 0.99, 1.00 | 0.074 |
| Age | 102 | 1.01 | 0.98, 1.04 | 0.5 |
|  | | | | |

In multivariable Fine and Gray regression for OHE development, liver transplantation and death were treated as a competing event. Abbr.: sHR, subdistribution hazard ratio; CI, confidence interval; NfL, neurofilament light chains; PSG, portosystemic gradient; MELD, model for end-stage liver disease; OHE, overt hepatic encephalopathy.

# Table S9: Multivariable Fine and Gray regression analysis for OHE development in the subgroup of patients with ascites as TIPS indication.

| **Characteristic** | **N** | **sHR** | **95% CI** | **p-value** |
| --- | --- | --- | --- | --- |
| GFAP | 102 | 1.00 | 1.00, 1.00 | 0.3 |
| PSG delta (%) | 102 | 1.02 | 0.99, 1.04 | 0.2 |
| MELD | 102 | 1.06 | 0.99, 1.14 | 0.073 |
| History of OHE |  |  |  |  |
| 0 | 73 | — | — |  |
| 1 | 29 | 1.80 | 0.92, 3.53 | 0.087 |
| Age | 102 | 1.01 | 0.99, 1.04 | 0.3 |
| Platelets (/nl) | 102 | 1.00 | 0.99, 1.00 | 0.2 |
|  | | | | |

In multivariable Fine and Gray regression for OHE development, liver transplantation and death were treated as a competing event. Abbr.: sHR, subdistribution hazard ratio; CI, confidence interval; GFAP, glial fibrillary acidic protein; PSG, portosystemic gradient; MELD, model for end-stage liver disease; OHE, overt hepatic encephalopathy.

# Table S10: Univariable Cox regression analysis for death/liver transplantation.

| **Characteristic** | **N** | **HR** | **95% CI** | **p-value** |
| --- | --- | --- | --- | --- |
| Age (years) | 144 | 1.03 | 1.00, 1.06 | 0.032 |
| Female sex | 144 | 0.55 | 0.26, 1.14 | 0.11 |
| PSG delta (%) | 144 | 1.00 | 0.99, 1.02 | 0.6 |
| TIPS diameter (mm) | 144 | 0.99 | 0.78, 1.24 | 0.9 |
| MELD | 144 | 1.16 | 1.08, 1.24 | <0.001 |
| FIPS | 144 | 3.03 | 1.91, 4.79 | <0.001 |
| History of OHE | 144 | 2.06 | 1.12, 3.78 | 0.020 |
| CHE | 124 | 1.67 | 0.84, 3.32 | 0.15 |
| PHES | 124 | 0.93 | 0.86, 1.00 | 0.064 |
| Sodium (mmol/L) | 142 | 0.90 | 0.85, 0.95 | <0.001 |
| Creatinine (mg/dL) | 144 | 1.60 | 1.27, 2.01 | <0.001 |
| Bilirubin (mg/dL) | 144 | 1.28 | 0.93, 1.76 | 0.14 |
| INR | 144 | 2.39 | 0.62, 9.28 | 0.2 |
| Albumin (g/L) | 144 | 0.92 | 0.88, 0.96 | <0.001 |
| CRP (mg/L) | 142 | 1.01 | 1.00, 1.02 | 0.2 |
| WBC (per nL) | 143 | 0.95 | 0.85, 1.07 | 0.4 |
| Hemoglobin (g/dL) | 143 | 0.82 | 0.70, 0.96 | 0.014 |
| Platelets (per nL) | 142 | 1.00 | 1.00, 1.00 | 0.9 |
| NfL (pg/mL) | 144 | 1.01 | 1.00, 1.01 | 0.028 |
| NfL above the median | 144 | 2.20 | 1.18, 4.13 | 0.014 |
| GFAP (pg/mL) | 144 | 1.00 | 1.00, 1.00 | 0.3 |
| GFAP above the median | 144 | 1.67 | 0.91, 3.08 | 0.10 |
| Lactulose | 144 | 1.16 | 0.63, 2.13 | 0.6 |
| Rifaximin | 144 | 0.71 | 0.39, 1.30 | 0.3 |
|  | | | | |

Abbr.: sHR, subdistribution hazard ratio; CI, confidence interval; PSG, portosystemic gradient; MELD, model for end-stage liver disease; FIPS, Freiburg index for post-TIPS survival, OHE, overt hepatic encephalopathy; CHE, covert hepatic encephalopathy; PHES, psychometric hepatic encephalopathy score; CRP, c-reactive protein; WBC, white blood cell count; NfL, neurofilament light chains; GFAP, glial fibrillary acidic protein.
